# The Dynamic Expression of Potential Mediators of Severe Acute Respiratory Syndrome Coronavirus 2 Cellular Entry in Fetal, Neonatal, and Adult Rhesus Monkeys

**DOI:** 10.3389/fgene.2020.607479

**Published:** 2021-01-18

**Authors:** Bangrong Cao, Liping Zhang, Huifen Liu, Shiqi Ma, Kun Mi

**Affiliations:** ^1^Radiation Oncology Key Laboratory of Sichuan Province, Sichuan Cancer Center, School of Medicine, Sichuan Cancer Hospital and Institute, University of Electronic Science and Technology of China, Chengdu, China; ^2^Department of Clinical Laboratory, Sichuan Provincial Maternity and Child Health Care Hospital, The Affiliated Women’s and Children’s Hospital of Chengdu Medical College, Chengdu, China

**Keywords:** SARS-CoV-2, Rhesus monkey, development, ACE2, *TMPRSS2*

## Abstract

The coronavirus disease 2019 (COVID-19) pandemic, induced by the pathogenic severe acute respiratory syndrome coronavirus 2 (SARS-CoV-2), has spread rapidly all over the world. There is considerable variability among neonates, children, and adults in the incidence of infection and severe disease following exposure to SARS-CoV-2. In our study, we analyzed the transcriptome data of primate animal model of Rhesus monkeys to evaluate the expression levels of possible SARS-CoV-2 receptors and proteases and immunologic features in the lungs, colons, livers, and brains at different developmental stages. Our results revealed that *ACE2* and *TMPRSS2* were highly expressed in neonates compared with other populations, which imply the high incidence of infection. Other potential receptors and Type II transmembrane serine proteases (TTSPs) and cathepsin of endosomal proteases also exhibited dynamic and differential expression patterns. The expression of receptors (*ACE2*, *BSG*, and *DPP4*) and proteases (*TMPRSS2*, *TMPRSS9*, *CTSL*, and *CTSB*) were highly correlated during lung development, suggesting the high susceptibility of the lungs. *TMPRSS9* was specifically highly expressed in the lungs and reached the highest level in neonates, similar to *TMPRSS2*. Moreover, the immune cell infiltration analysis revealed immunity immaturity in neonates, implying the association with the mild or moderate type of COVID-19. The results might help researchers design protective and therapeutic strategies for COVID-19 in populations at different ages.

## Introduction

The coronavirus disease 2019 (COVID-19) pandemic, caused by the novel coronavirus severe acute respiratory syndrome coronavirus 2 (SARS-CoV-2), has spread rapidly all over the world with significant public health concerns. By September 14, 2020, the COVID-19 pandemic has resulted in approximately 29 million cases and over 9,22,000 deaths worldwide. In China, there have been over 90,000 confirmed cases and 4,700 deaths^1^. Despite the worldwide spread, the epidemiological and clinical patterns of the COVID-19 remain largely unclear among neonates and children. The epidemiological characteristics of pediatric patients with COVID-19 in China have indicated that young children, particularly infants, were vulnerable to SARS-CoV-2 infection (Dong et al., 2020). Moreover, neonatal cases showed that similar to older children, most neonates with COVID-19 were asymptomatic (20%) or had mild (48%) and moderate (20%) signs of clinical infection (Liguoro et al., 2020). Similarly, in the United States, 27% of the pediatric cases that tested positive were asymptomatic, while only 7% of adults were asymptomatic (Rawat et al., 2020). A higher proportion of asymptomatic carriers in neonates and children could make them effective carriers to spread the viruses (Gandhi et al., 2020). There is an urgent need to identify the molecular mechanisms that mediate viral entry, propagation, and viral susceptibility in this population.

The types of human cells and tissues targeted by SARS-CoV-2, its potential receptors, and associated mediators for cellular entry are still largely unknown. Both SARS-CoV-2 and SARS-CoV use cell membrane-bound angiotensin-converting enzyme 2 (*ACE2*) as one of the main receptors for entry into host cells with the help of associated proteases such as transmembrane protease serine 2 (*TMPRSS2*) for S protein priming (Hoffmann et al., 2020; Radzikowska et al., 2020). *CD147* (*BSG*) has been recently shown to act as a receptor for SARS-CoV-2 in cell lines of epithelial origin, which is also a putative receptor for SARS-CoV-2, HIV-1, and measles for entry to host cells (Radzikowska et al., 2020). *ACE2* is a human homology of *ACE* (Tipnis et al., 2000). The expression of *ACE2* is dynamically changed with age; it was reported that *ACE2* mRNA is expressed in the lungs and trachea shortly after birth, downregulated during childhood, and expressed at high levels in late adulthood again in alveolar epithelial cells (Inde et al., 2020). Other potential receptors related to coronavirus include *DPP4*, *ANPEP*, *ENPEP*, *CD209*, and *CLEC4G* (Bassendine et al., 2020; Qi et al., 2020). The host cell proteases, Type II transmembrane serine proteases (TTSPs), and cathepsin family of endosomal proteases, which play a crucial role in the entry process for S protein priming, are essential for viral infectivity (Simmons et al., 2013). TTSPs activate a wide range of viruses such as SARS, Middle East respiratory syndrome (MERS) coronaviruses, human metapneumoviruses, and influenza viruses (Shin and Seong, 2017). There are 17 members of human TTSPs identified so far (Bugge et al., 2009). The cathepsin family of endosomal proteases is required for proteolytic processing of several viruses during entry into host cells (Johnson et al., 2009). Cell culture studies demonstrated that activation of SARS and MERS coronavirus could be accomplished by the endosomal cysteine proteases, cathepsin L (*CTSL*), and cathepsin B (*CTSB*) (Zhou et al., 2015). Whether these mediators are dynamically and deferentially expressed in the developmental process of fetuses, neonates, and adults could elucidate the pathophysiology of these populations at different age status.

It has been reported that compared with those of adult patients, the clinical manifestations of pediatric COVID-19 cases were generally less severe (Liguoro et al., 2020). Case series of hospitalized pediatric patients in Wuhan, China, indicated that systemic inflammation rarely occurred in pediatric patients with COVID-19, while aggravated inflammatory responses were frequently observed in adults with COVID-19 (Wu H. et al., 2020). The innate immune and adaptive immune cells develop and mature during fetal life and after birth. Their functions are weak in newborns and young children than in later life and resulted in diminished cytokine responses as compared with those in adults (Simon et al., 2015). The immunologic features related to the clinical outcomes of COVID-19 in fetuses, neonates, and children may further explain this manifestation from the developmental perspective.

Since there is lack of data of the viral susceptibility of SARS-CoV-2 in fetuses and neonates of human beings, we utilized the primate animal model of Rhesus monkeys to evaluate the expression of SARS-CoV-2-related receptors, associated proteases, and immunologic features in different developmental stages and different organs. Our study gives a further perspective of infection potential of SARS-CoV-2 and its impact on fetal, neonatal, and adult Rhesus monkeys on the gene expression level.

## Materials and Methods

### Rhesus Macaques and Gene Expression Data

Gene expression data of developmental tissues from Rhesus monkeys were obtained from previously published studies (Xu et al., 2016; Yu et al., 2016). The data were provided in the form of normalized gene expression levels of reads per kilobase of transcript per million mapped reads (RPKM). The dataset included tissues of the lungs, liver, brain, and colon mucosa from Rhesus macaques at different developmental stages. The development time points included early stage (45–70 days, *n* = 2), middle stage (100 days, *n* = 3), antenatal stage (137–163 days, *n* = 3), neonatal stage (post born 4–7 days, *n* = 3), and adult (post born 5–8 years, *n* = 3). The animal study was approved by the institutional ethics committee as declared in the original literature (Yu et al., 2016).

### Gene Expression Analysis

Gene expression levels of 29 mediators potentially involved in the SARS-CoV-2 infection process were comprehensive evaluated. These genes include confirmed receptors for SARS-CoV-2 (*ACE2* and *BSG*) (Hoffmann et al., 2020; Radzikowska et al., 2020), potential receptors for SARS-CoV-2 (*DPP4*, *ANPEP*, *ENPEP*, *CD209*, *CLEC4G*, and *ACE*) (Bassendine et al., 2020; Qi et al., 2020; Tipnis et al., 2000), protease mediating SARS-CoV-2 entry (*TMPRSS2*) (Hoffmann et al., 2020), other potential proteases of the transmembrane protease serine families mediating SARS-CoV-2 entry (*TMPRSS11D*, *TMPRSS11E*, *TMPRSS11A*, *TMPRSS11B*, *TMPRSS1*, *TMPRSS2*, *TMPRSS3*, *TMPRSS4*, *TMPRSS5*, *TMPRSS6*, *TMPRSS7*, *TMPRSS9*, *TMPRSS10*, *TMPRSS13*, *TMPRSS15*, and *ST14*) (Bugge et al., 2009; Simmons et al., 2013; Shin and Seong, 2017), and the potential proteases of the cathepsins mediating SARS-CoV-2 entry (*CTSB*, *CTSL*, *CTSS*, *CTSC*, and *CTSD*) (Johnson et al., 2009; Zhou et al., 2015). *TMPRSS11C* was excluded due to its absence in the transcriptome data. The spatiotemporal expression pattern of these genes during monkey development were illustrated by heatmap and tendency chart.

### Immunologic Characteristics

The immunologic characteristics of developmental tissues were calculated by using the MCP-counter, an estimator for abundance of tissue infiltrating immune cells by gene expression profiles (Becht et al., 2016). The R package “MCPcounter” was employed to estimate the expression scores of eight immune cells, including T cells, CD8 + T cells, B lineage, NK cells, cytotoxic lymphocytes, monocytic lineage, myeloid dendritic cells, and neutrophils. In addition, this study also analyzed the gene expression levels of 15 cytokines, which may be involved in cytokine release syndrome in patients with severe COVID-19 (Guzik et al., 2020; Pedersen and Ho, 2020; Wang et al., 2020; Zhang H. et al., 2020).

### Statistical Analysis

Gene expression profiles of candidate genes or expression scores of immune cells were visualized by using the R package “pheatmap” (Version 1.0.10). Expression trends of specified genes along with developmental time were fitted by the Lowess method using the R package “ggplot2” (Version 3.1.0). The Pearson correlation analysis was employed to estimate the gene expression correlation among candidate genes with the use of R package “corrplot” (Version 0.84). The statistical differences of immune cell scores or gene expression levels among different developmental stages were calculated by using unpaired Student’s *t*-test. Multiple tests were adjusted by using the Benjamini and Hochberg method. All statistical analyses were performed by using the R software (Version 3.5.1). A *p* < 0.05 was considered as statistically significant.

## Results

### Spatiotemporal Gene Expression of Severe Acute Respiratory Syndrome Coronavirus 2 Infection-Related Mediators During Rhesus Monkey Development

**FIGURE 1:**
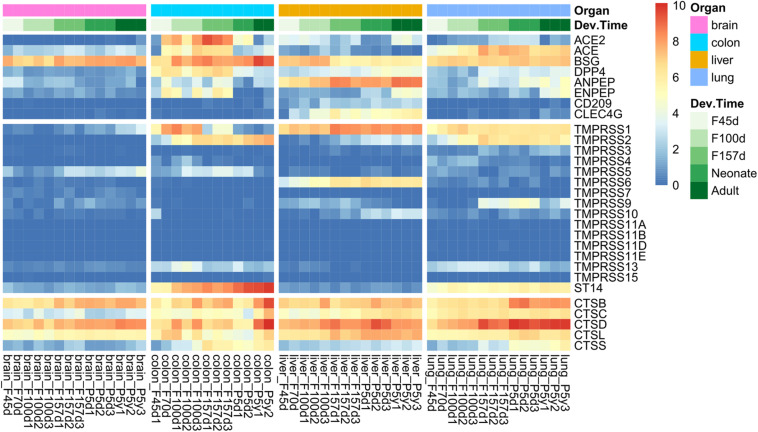
Spatiotemporal expression of severe acute respiratory syndrome coronavirus 2 (SARS-CoV-2) infection mediators in Rhesus monkey development. The heatmap represents gene expression value [log2(RPKM + 1)] of tissues from four organs at different developmental stages, including early (F45d–F70d), middle (F100d), and antenatal (F157d) stages of fetuses, neonates (post born 5–7 days), and adults (post born 5–7 years).

The overview of spatiotemporal expression patterns of SARS-CoV-2 infection-associated genes among four organs is depicted in Figure 1. Considering the SARS-CoV-2 receptors and transmembrane protease serine families, tissues from the colon, liver, and lungs exhibited similar gene expression patterns, which were quite different from those of the brain (Figure 1, top and middle panels). The brain tissues had very low expression levels of these genes, except for *BSG*. However, the expression patterns of cathepsins were more likely to be consistent among the four organs (Figure 1, bottom panel). We found that *ACE*, *TMPRSS2*, *TMPRSS13*, *ST14*, and *CTSS* were specifically expressed in lung and colon tissues (Figure 1). Very interestingly, the highest expression level of *ACE2* was observed in colon tissues, as compared with that in the lungs and livers. There were also organ-specifically expressed genes, including *TMPRSS9* in the lungs, *CLEC4G* and *TMPRSS6* in the liver. Moreover, both *ACE2* and *TMPRSS2* were nearly not expressed in brain tissues (Figure 1).

### Expression Trend of Specific Genes During Lung and Colon Development

**FIGURE 2:**
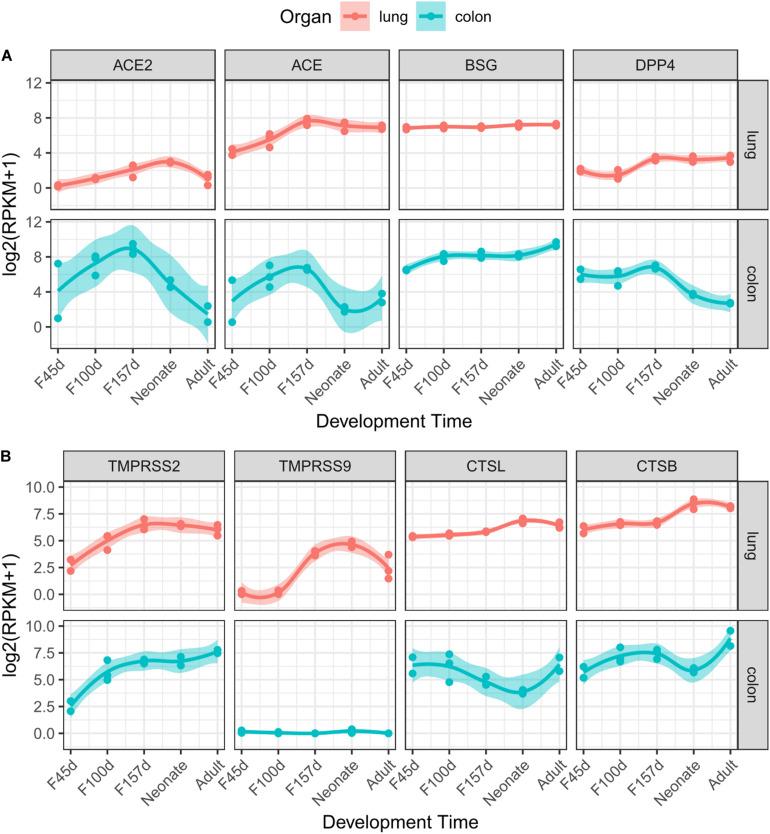
Dynamic expression of eight genes during lung and colon development. Gene expression value of receptors **(A)** and proteases **(B)** along with developmental times are presented for the lungs (red) or colon (cyan). The trend curves were fitted by using LOESS method, with standard errors, as shown by shadows.

*ACE2* has been demonstrated to be the receptor of SARS-CoV-2 on target cells according to current evidence (Hoffmann et al., 2020; Li et al., 2020; Radzikowska et al., 2020; Zhang H. et al., 2020). Although *ACE2* was expressed at relatively low levels in lung tissues (Figure 1), it was gradually elevated from fetal periods to neonatal stage (Figure 2A). At the neonatal stage, *ACE2* reached the highest level, which declined subsequently in the adult lungs. Similar patterns were found in the expression of *ACE* and *DPP4*, which increased from early fetal stages to late fetal stages and stabilized in the neonatal and adult stages. In particular, the expression of *BSG* was at relatively high levels at all developmental stages of the lungs. *TMPRSS2*, which helps cellular entry of virus, was expressed at a low level in early fetal lungs and was significantly elevated during lung development (Figure 2B). A similar expression trend was observed for *TMPRSS9* but declined at the adult stage. The expression levels of *CTSL* and *CTSB* were increased in the fetal–neonatal periods and were slightly declined at the adult stage (Figure 2B). Collectively, these data suggested that most receptors and proteases involved in SARS-CoV-2 infection were consistently elevated during lung development and reached the highest level at the neonatal stage.

Some patients with COVID-19 were reported to present diarrhea symptoms (D’Amico et al., 2020), so we explored the gene expression patterns in the colon mucosa. However, most receptor genes (*ACE2*, *ACE*, and *DPP4*) were increased in the fetal periods but then dominantly decreased after delivery (Figure 2A). This expression pattern was quite different from that of the proteases, which showed an increasing trend (*TMPRSS2*) or a “V” shape (*CTSL* and *CTSB*) along with colon development (Figure 2B).

### Expression Correlation of Specific Genes in the Development of Four Organs

**FIGURE 3:**
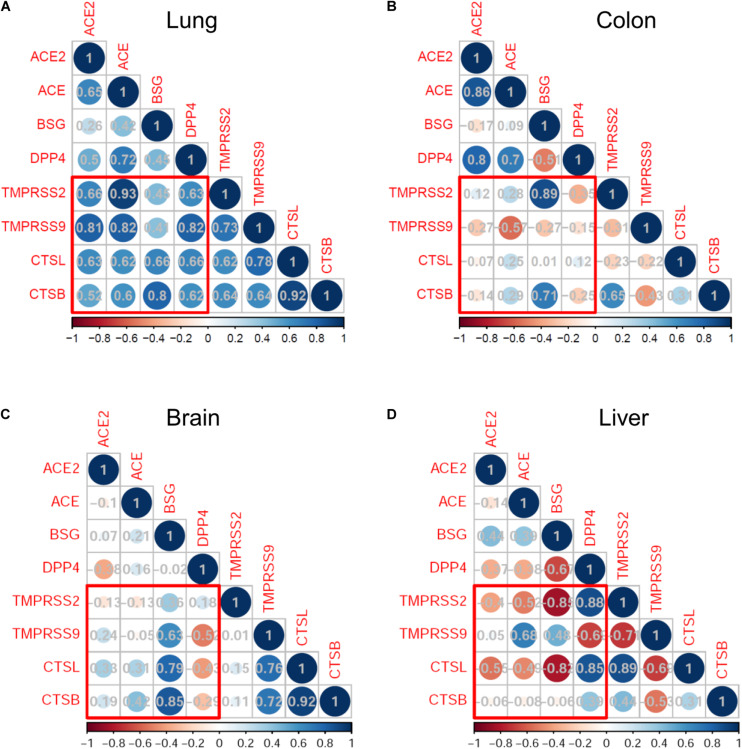
Correlation analysis of severe acute respiratory syndrome coronavirus 2 (SARS-CoV-2) infection mediators in monkey development. Correlation map represents the Pearson correlation coefficient between each pair of genes. The bottom bars indicate the color legends of the Pearson correlation coefficient *r*. Results are shown in the lungs **(A)**, colon **(B)**, brain **(C)**, and liver **(D)**.

We next analyzed the expression correlation of SARS-CoV-2 infection-associated genes in the development by using the Pearson correlation method. In lung development, the expression of putative SARS-CoV-2 receptors (*ACE2*, *ACE*, *BSG*, and *DPP4*) and proteases (*TMPRSS2*, *TMPRSS9*, *CTSL*, and *CTSB*) was highly correlated, with coefficient *r* more than 0.6 in most cases (Figure 3A). Particularly, the expression of *ACE2* was significantly correlated with that of *TMPRSS2* (*r* = 0.66, *p* = 0.01), *TMPRSS9* (*r* = 0.81, *p* < 0.001) and *CTSL* (*r* = 0.63, *p* = 0.015). Similar correlation patterns were observed for *ACE* and *DPP4*, both of which were significantly correlated with the four proteases (Figure 3A). The highly correlated gene expression of receptors and proteases putatively involved in SARS-CoV-2 infection suggested that the lungs are highly susceptible to SARS-CoV-2.

On the other hand, the expression correlations between receptors and proteases in the colons, brains, and livers were much weaker than those observed in the lungs (Figures 3B–D). Especially, there was no statistically positive correlation of *ACE2* expression with any of the four proteases in the three organs.

### Immunologic Features of Rhesus Monkeys at Different Developmental Stages

**FIGURE 4:**
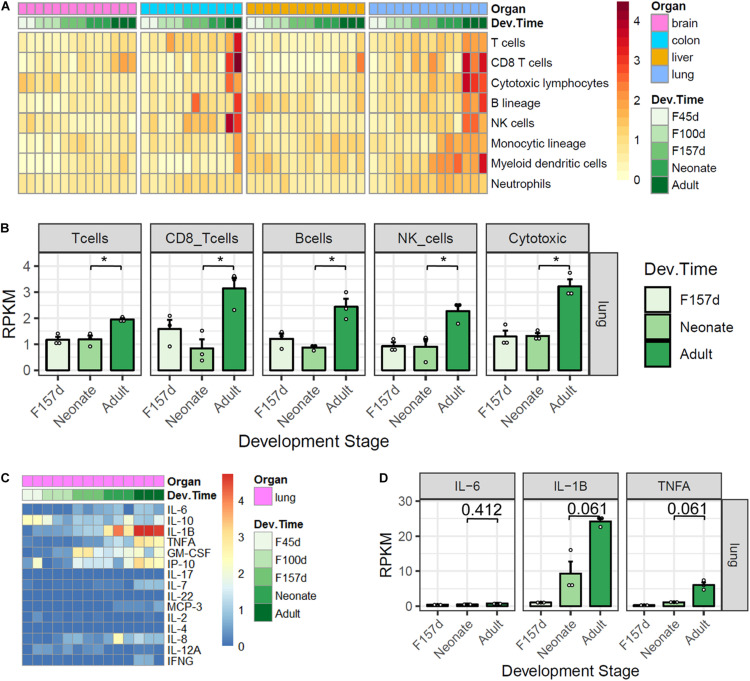
Immune features of four organs in monkey development. **(A)** Gene expression scores of immune cell populations in developmental tissues. **(B)** Five immune cells are elevated in adult lungs. **(C)** Expression profiles of 15 cytokines. **(D)** IL-1β and TNF-α are elevated in adult lungs. *P*-value was calculated by unpaired Student’s *t*-test and adjusted by using the Benjamini and Hochberg method for multiple tests. Note: *, adjusted *p* < 0.05.

In spite of the higher expression levels of *ACE2* and *TMPRSS2* in neonates compared with adults, the clinical manifestations of pediatric COVID-19 cases were generally less severe. We then sought to explore the differences in immune characteristics between the two populations. By using the MCP-counter, we investigated the immune features of innate and adoptive immune cells in developmental tissues among four organs. Results showed that the majority of immune cells were highly infiltrated in the lungs and colons, especially in adults (Figure 4A). In lung tissues, the expression scores of T cells, CD8 + T cells, B cells, NK cells, and cytotoxic lymphocytes were significantly higher in adults than in neonates (Figure 4B, all adjusted *p* < 0.05). However, there were no statistically significant differences for the immune profiles between antenatal fetuses and neonates. The expression profiles of 15 cytokines related to cytokine release syndrome were also explored in lung development (Figure 4C). Although interleukin (IL)-6 expression levels were comparable between adults and neonates, the expression levels of IL-1β (adjusted *p* = 0.061) and TNF-α (adjusted *p* = 0.061) tended to be higher in adults than in neonates (Figure 4D).

## Discussion

The mechanisms about SARS-CoV-2 cellular entry and epidemiological features of COVID-19 disease need to be continuously explored. However, current data have been reported mainly from adult patients with COVID-19, while the infection patterns of children, especially fetuses and neonates, still remain unclear. In this study, we explored the expression levels of SARS-CoV-2 cellular entry-related receptors and protease genes in the lungs, colons, livers, and brains of Rhesus monkey in different developmental stages of F45d–F70d (early stage), F100d (middle stage), and F157d (antenatal stage) of fetuses, neonates (post born 5–7 days), and adults (post born 5–7 years).

The roles of *ACE2* and *TMPRSS2* have been confirmed in SARS-CoV-2 cellular entry activities (Hoffmann et al., 2020; Ziegler et al., 2020). However, for the first time, our data pointed out that these two partners have consistent trends during the lung development of Rhesus monkeys, which kept rising from fetuses to neonates and then declined in adults, implying the highest viral susceptibility in the population of neonates. Meanwhile, the expression patterns were organ specific since tissues from the colon, liver, and lungs exhibited similar gene expression patterns, while there was almost no expression of *ACE2* and *TMPRSS2* in the brains. There is evidence that SARS-CoV-2 might have a different way of infection to the cerebral nervous system (Qiao et al., 2020). Moreover, the highest expression level of *ACE2* was observed in colon tissues, which may explain the presenting symptom of diarrhea in COVID-19 patients. An increasing number of diarrhea cases are reported (D’Amico et al., 2020). In COVID-19 patients, SARS-CoV-2 was reported to be detected in feces, suggesting the fecal–oral transmission (Wu Y. et al., 2020). The expression of *ACE2* exhibits heterogeneity; it was reported that *ACE2* gene expression in the nasal epithelium and the lung airways was lower in children than in adults (Bunyavanich et al., 2020; Saheb Sharif-Askari et al., 2020) and that *ACE2* mRNA is expressed in the lungs and trachea shortly after birth, downregulated during childhood, and expressed at high levels in late adulthood again in alveolar epithelial cells (Inde et al., 2020). Considering the high viral susceptibility and frequent defecation in infants, there should be more careful nursing measures for this population under COVID-19 risk.

However, it is still not clear whether other coronavirus-related receptors and enzymes could also activate SARS-CoV-2 for spread in target host cells. We studied a broad range of receptors including *DPP4*, *ANPEP*, *ENPEP*, *CD209*, and *CLEC4G*, which have been reported to be involved in the cellular entry of coronaviruses (Bassendine et al., 2020; Qi et al., 2020). Meanwhile, we investigated the dynamic expression levels of two major protease families, TTSPs, and cathepsin. The findings of our study showed that *TMPRSS2*, *TMPRSS13*, *ST14*, and *CTSS* exhibited specific high expression levels in the lungs and colons, which were preferentially targeted by SARS-CoV-2, suggesting their possible involvement in virus cellular entry to these organs. During lung development, the expression of receptors (*ACE2*, *BSG*, and *DPP4*) and proteases (*TMPRSS2*, *TMPRSS9*, *CTSL*, and *CTSB*) were highly correlated, implying the high susceptibility to SARS-CoV-2 of the lungs.

*TMPRSS9* has been identified as a candidate gene that may be involved in biological pathways leading to respiratory symptoms (Zeng et al., 2019). Our results indicated that *TMPRSS9* was specifically highly expressed in the lungs and reached the highest level in Rhesus monkeys at the neonatal stage like *TMPRSS2*. The Pearson correlation coefficient analysis indicated that expression of *ACE2* was significantly correlated with that of *TMPRSS9* (*r* = 0.81, *p* < 0.001). Moreover, for COVID-19 patients, except for fever (32%) and feeding intolerance (24%), dyspnea was the most common reported sign in neonatal age (40%) (Liguoro et al., 2020). Therefore, whether *TMPRSS9* might play a role in SARS-CoV-2 lung infection processes needs to be further explored in the future.

The clinical manifestations of pediatric COVID-19 cases were generally less severe than those of adults (Liguoro et al., 2020), while information on immune features associated with disease severity is insufficient. It has been reported that SARS-CoV-2 caused more severe interstitial pneumonia in old Rhesus monkeys than that in young monkeys (Yu et al., 2020). We compared the immunologic features of immune cells subgroups and cytokines among fetus, neonate, and adult populations at different developmental stages of Rhesus monkeys. The immune cell subgroup analysis showed that the expression scores of T cells, CD8 + T cells, B cells, NK cells, and cytotoxic lymphocytes in the lungs were significantly higher in adults than in neonates. The decreased expression of immune cells might protect them from serious pulmonary consequences in which immune cells release large amounts of proinflammatory cytokines that could cause serious damage to the host. These results revealed the immunity immaturity in neonates may be directly associated with the mild or moderate type of COVID-19 and make them efficient carriers with a high incidence of asymptomatic or mildly symptomatic infection.

The weak functions of innate immune and adaptive immune cells in newborns and young children could result in diminished cytokine responses as compared with those in adults (Simon et al., 2015). We analyzed 15 cytokines related to cytokine release syndrome in lung development of Rhesus monkeys and found that compared with those in neonates, levels of proinflammatory cytokine tumor necrosis factor α and IL-1β were significantly elevated in adults, and levels of IL-6 were comparable between adults and neonates, which might in part explain the less severe inflammatory responses in pediatric COVID-19 patients compared with adults. As for IL-6, it was reported that their levels were unchanged in moderate COVID-19 cases compared with mild cases (Wu H. et al., 2020), while other reports revealed that elevated IL-6 levels were observed during COVID-19 progression (Pedersen and Ho, 2020; Zhang C. et al., 2020). These results suggested that the regulation of IL-6 in COVID-19 progression is complicated and needs further exploration.

Although there was no confirmed conclusion of intrauterine transmission of SARS-CoV-2 from infected mothers with COVID-19 to their fetuses, possible vertical transmission of SARS-CoV-2 was reported in which a neonate born to a mother with COVID-19 was found to have elevated IgM and IgG antibody levels and abnormal cytokine test results 2 h after birth (Dong et al., 2020). Since IgM antibodies could not be transferred to the fetus via the placenta (Woo et al., 2004), the IgM antibody result suggests that the neonate was infected *in utero*. Our results could provide clinical management advice for pregnant women with suspected or confirmed COVID-19 infection by evaluating their miscarriage risk and the infection risk in fetuses due to the dynamic expression levels of possible SARS-CoV-2-related receptors and proteases. Moreover, the high risk of infection of neonates implied by the highest expression level of *ACE2* and *TMPRSS2* suggests that neonates should get the COVID-19 vaccine as soon as they were born if the vaccines are available.

We utilized the primate animal model of Rhesus monkeys to evaluate the dynamic expression of potential mediators of SARS-CoV-2 cellular entry since there is lack of data of the viral susceptibility of SARS-CoV-2 in fetuses and neonates of human beings. Although there are differences between these two species, these suggestive results have important implications in our fighting against COVID-19 pandemic. This study is limited by the small sample size, especially colon samples, and lack of data from young Rhesus monkeys at age status between neonates and adults. The lack of gender information also limits our research in the study of whether the expression of certain genes is affected by gender. Further study could include more samples of Rhesus monkeys at different ages of different genders and different organs to confirm this preliminary conclusion and reveal more details in the cellular entry mechanisms, infection patterns of SARS-CoV-2, and epidemiology and pathophysiology of COVID-19.

## Data Availability Statement

The original contributions presented in the study are included in the article/Supplementary Material, further inquiries can be directed to the corresponding author/s.

## Ethics Statement

The animal study was reviewed and approved by the institutional ethics committee as declared in the original literature previously (Yu et al., 2016).

## Author Contributions

KM conceptualized and designed the study, drafted the initial manuscript, and reviewed and revised the manuscript. BC designed the study, carried out the initial analyses, interpreted the results, and reviewed and revised the manuscript. LZ collected the data, interpreted the results, and reviewed and revised the manuscript. HL and SM coordinated and supervised data collection and reviewed and revised the manuscript. All authors approved the final manuscript as submitted and agreed to be accountable for all aspects of the work.

## Conflict of Interest

The authors declare that the research was conducted in the absence of any commercial or financial relationships that could be construed as a potential conflict of interest.

## Footnotes

1 https://www.who.int/emergencies/diseases/novel-coronavirus-2019/situation-reports

## References

[B1] Bassendine M. F. Bridge S. H. McCaughan G. W. Gorrell M. D. (2020). COVID-19 and comorbidities: a role for dipeptidyl peptidase 4 (DPP4) in disease severity? *J. Diabetes* 12 649–658. 10.1111/1753-0407.13052 32394639

[B2] Becht E. Giraldo N. A. Lacroix L. Buttard B. Elarouci N. Petitprez F. (2016). Estimating the population abundance of tissue-infiltrating immune and stromal cell populations using gene expression. *Genome Biol.* 17:218. 10.1186/S13059-016-1070-5 27765066 PMC5073889

[B3] Bugge T. H. Antalis T. M. Wu Q. (2009). Type II transmembrane serine proteases. *J. Biol. Chem.* 284 23177–23181. 10.1074/jbc.R109.021006 19487698 PMC2749090

[B4] Bunyavanich S. Do A. Vicencio A. (2020). Nasal gene expression of angiotensin-converting Enzyme 2 in children and adults. *JAMA J. Am. Med. Assoc.* 323 2427–2429. 10.1001/jama.2020.8707 PMC7240631 32432657

[B5] D’Amico F. Baumgart D. C. Danese S. Peyrin-Biroulet L. (2020). Diarrhea during COVID-19 infection: pathogenesis, epidemiology, prevention and management. *Clin. Gastroenterol. Hepatol.* 18 1663–1672. 10.1016/j.cgh.2020.04.001 32278065 PMC7141637

[B6] Dong L. Tian J. He S. Zhu C. Wang J. Liu C. (2020). Possible vertical transmission of SARS-CoV-2 from an infected mother to her newborn. *JAMA J. Am. Med. Assoc.* 323 1846–1848. 10.1001/jama.2020.4621 32215581 PMC7099527

[B7] Gandhi M. Yokoe D. S. Havlir D. V. (2020). Asymptomatic Transmission, the Achilles’ Heel of Current Strategies to Control Covid-19. *N. Engl. J. Med.* 382 2158–2160. 10.1056/nejme2009758 32329972 PMC7200054

[B8] Guzik T. J. Mohiddin S. A. Dimarco A. Patel V. Savvatis K. Ber M.-B. (2020). COVID-19 and the cardiovascular system: implications for risk assessment, diagnosis, and treatment options. *Cardiovasc. Res.* 116 1666–1687. 10.1093/CVR/CVAA106 32352535 PMC7197627

[B9] Hoffmann M. Kleine-Weber H. Schroeder S. Krüger N. Herrler T. Erichsen S. (2020). SARS-CoV-2 cell entry depends on ACE2 and TMPRSS2 and is blocked by a clinically proven protease inhibitor. *Cell* 181 271–280.e8. 10.1016/j.cell.2020.02.052 32142651 PMC7102627

[B10] Inde Z. Yapp C. Joshi G. N. Spetz J. Fraser C. Deskin B. (2020). Age-dependent regulation of SARS-CoV-2 cell entry genes and cell death programs correlates with COVID-19 disease severity. *bioRxiv* [Preprint]. 10.1101/2020.09.13.276923 PMC8373124 34407940

[B11] Johnson E. M. Doyle J. D. Wetzel J. D. McClung R. P. Katunuma N. Chappell J. D. (2009). Genetic and pharmacologic alteration of cathepsin expression influences reovirus pathogenesis. *J. Virol.* 83 9630–9640. 10.1128/jvi.01095-09 19640986 PMC2748054

[B12] Li G. He X. Zhang L. Ran Q. Wang J. Xiong A. (2020). Assessing ACE2 expression patterns in lung tissues in the pathogenesis of COVID-19. *J. Autoimmun.* 112:102463. 10.1016/j.jaut.2020.102463 32303424 PMC7152872

[B13] Liguoro I. Pilotto C. Bonanni M. Ferrari M. E. Pusiol A. Nocerino A. (2020). SARS-COV-2 infection in children and newborns: a systematic review. *Eur. J. Pediatr.* 179 1029–1046. 10.1007/s00431-020-03684-7 32424745 PMC7234446

[B14] Pedersen S. F. Ho Y. C. (2020). SARS-CoV-2: a storm is raging. *J. Clin. Invest.* 130 2202–2205. 10.1172/JCI137647 32217834 PMC7190904

[B15] Qi F. Qian S. Zhang S. Zhang Z. (2020). Single cell RNA sequencing of 13 human tissues identify cell types and receptors of human coronaviruses. *Biochem. Biophys. Res. Commun.* 526 135–140. 10.1016/j.bbrc.2020.03.044 32199615 PMC7156119

[B16] Qiao J. Li W. Bao J. Peng Q. Wen D. Wang J. (2020). The expression of SARS-CoV-2 receptor ACE2 and CD147, and protease TMPRSS2 in human and mouse brain cells and mouse brain tissues. *Biochem. Biophys. Res. Commun.* 533 867–871. 10.1016/j.bbrc.2020.09.042 33008593 PMC7489930

[B17] Radzikowska U. Ding M. Tan G. Zhakparov D. Peng Y. Wawrzyniak P. (2020). Distribution of ACE2, CD147, CD26 and other SARS-CoV-2 associated molecules in tissues and immune cells in health and in asthma, COPD, obesity, hypertension, and COVID-19 risk factors. *Allergy* 10.1111/all.14429 [Epub ahead of print]. PMC7300910 32496587

[B18] Rawat M. Chandrasekharan P. Hicar M. D. Lakshminrusimha S. (2020). COVID-19 in Newborns and Infants-Low Risk of Severe Disease: Silver Lining or Dark Cloud? *Am. J. Perinatol.* 37 845–849. 10.1055/s-0040-1710512 32380565 PMC7356082

[B19] Saheb Sharif-Askari N. Saheb Sharif-Askari F. Alabed M. Temsah M. H. Al Heialy S. Hamid Q. (2020). Airways expression of SARS-CoV-2 receptor, ACE2, and TMPRSS2 Is lower in children than adults and increases with smoking and COPD. *Mol. Ther. Methods Clin. Dev.* 18 1–6. 10.1016/j.omtm.2020.05.013 32537478 PMC7242205

[B20] Shin W. J. Seong B. L. (2017). Type II transmembrane serine proteases as potential target for anti-influenza drug discovery. *Expert. Opin. Drug Discov.* 12 1139–1152. 10.1080/17460441.2017.1372417 28870104

[B21] Simmons G. Zmora P. Gierer S. Heurich A. Pöhlmann S. (2013). Proteolytic activation of the SARS-coronavirus spike protein: cutting enzymes at the cutting edge of antiviral research. *Antiviral Res.* 100 605–614. 10.1016/j.antiviral.2013.09.028 24121034 PMC3889862

[B22] Simon A. K. Hollander G. A. McMichael A. (2015). Evolution of the immune system in humans from infancy to old age. *Proc. R. Soc. B Biol. Sci.* 282:20143085. 10.1098/rspb.2014.3085 26702035 PMC4707740

[B23] Tipnis S. R. Hooper N. M. Hyde R. Karran E. Christie G. Turner A. J. (2000). A human homolog of angiotensin-converting enzyme: cloning and functional expression as a captopril-insensitive carboxypeptidase. *J. Biol. Chem.* 275 33238–33243. 10.1074/jbc.M002615200 10924499

[B24] Wang J. Jiang M. Chen X. Montaner L. J. (2020). Cytokine storm and leukocyte changes in mild versus severe SARS-CoV-2 infection: review of 3939 COVID-19 patients in China and emerging pathogenesis and therapy concepts. *J. Leukoc. Biol.* 108 17–41. 10.1002/JLB.3COVR0520-272R 32534467 PMC7323250

[B25] Woo P. C. Y. Lau S. K. P. Wong B. H. L. Tsoi H. W. Fung A. M. Y. Chan K. H. (2004). Detection of specific antibodies to severe acute respiratory syndrome (SARS) Coronavirus nucleocapsid protein for serodiagnosis of SARS coronavirus pneumonia. *J. Clin. Microbiol.* 42 2306–2309. 10.1128/JCM.42.5.2306-2309.2004 15131220 PMC404667

[B26] Wu H. Zhu H. Yuan C. Yao C. Luo W. Shen X. (2020). Clinical and immune features of hospitalized pediatric patients with Coronavirus disease 2019 (COVID-19) in Wuhan, China. *JAMA Netw. Open* 3:e2010895. 10.1001/jamanetworkopen.2020.10895 32492165 PMC7272117

[B27] Wu Y. Guo C. Tang L. Hong Z. Zhou J. Dong X. (2020). Prolonged presence of SARS-CoV-2 viral RNA in faecal samples. *Lancet Gastroenterol. Hepatol.* 5 434–435. 10.1016/S2468-1253(20)30083-2 32199469 PMC7158584

[B28] Xu J. Feng L. Han Z. Li Y. Wu A. Shao T. (2016). Extensive ceRNA-ceRNA interaction networks mediated by miRNAs regulate development in multiple rhesus tissues. *Nucleic Acids Res.* 44 9438–9451. 10.1093/nar/gkw587 27365046 PMC5100587

[B29] Yu P. Qi F. Xu Y. Li F. Liu P. Liu J. (2020). Age-related rhesus macaque models of COVID-19. *Anim. Model. Exp. Med.* 3 93–97. 10.1002/ame2.12108 32318665 PMC7167234

[B30] Yu X. Feng L. Han Z. Wu B. Wang S. Xiao Y. (2016). Crosstalk of dynamic functional modules in lung development of rhesus macaques. *Mol. Biosyst.* 12 1342–1349. 10.1039/c5mb00881f 26923754

[B31] Zeng X. Vonk J. M. van der Plaat D. A. Faiz A. Paré P. D. Joubert P. (2019). Genome-wide interaction study of gene-by-occupational exposures on respiratory symptoms. *Environ. Int.* 122 263–269. 10.1016/j.envint.2018.11.017 30449631

[B32] Zhang C. Wu Z. Li J. W. Zhao H. Wang G. Q. (2020). Cytokine release syndrome in severe COVID-19: interleukin-6 receptor antagonist tocilizumab may be the key to reduce mortality. *Int. J. Antimicrob. Agents* 55:105954. 10.1016/j.ijantimicag.2020.105954 32234467 PMC7118634

[B33] Zhang H. Rostami M. R. Leopold P. L. Mezey J. G. O’Beirne S. L. Strulovici-Barel Y. (2020). Expression of the SARS-CoV-2 ACE2 receptor in the Human Airway Epithelium. *Am. J. Respir. Crit. Care Med.* 202 219–229. 10.1164/rccm.202003-0541OC 32432483 PMC7365377

[B34] Zhou Y. Vedantham P. Lu K. Agudelo J. Carrion R. Nunneley J. W. (2015). Protease inhibitors targeting coronavirus and filovirus entry. *Antiviral Res.* 116 76–84. 10.1016/j.antiviral.2015.01.011 25666761 PMC4774534

[B35] Ziegler C. G. K. Allon S. J. Nyquist S. K. Mbano I. M. Miao V. N. Tzouanas C. N. (2020). SARS-CoV-2 receptor ACE2 is an interferon-stimulated gene in human airway epithelial cells and is detected in specific cell subsets across tissues. *Cell* 181 1016–1035.e19. 10.1016/j.cell.2020.04.035 32413319 PMC7252096

