# Supplementary material for: The Dynamic Expression of Potential Mediators of Severe Acute Respiratory Syndrome Coronavirus 2 Cellular Entry in Fetal, Neonatal, and Adult Rhesus Monkeys
Source: Front Genet. 2021 Jan 18;11:607479. doi: 10.3389/fgene.2020.607479 (PMC7848180; doi:10.3389/fgene.2020.607479)
Supplement: Supplementary file 1 [file Table_1.DOC]

library(pheatmap)

library(RColorBrewer)

options(stringsAsFactors=F)

rm(list = ls());

##01###############################################for gene analysis

##data input

data = read.table("ACE2_Immu_44genes_RPKM.txt",

header=T, row.names=1, sep="\t", quote="")

sub.Data = data.matrix(data[1:29,])

pheno = read.table("Monkey_phenotype.txt",

header=T, sep="\t", quote="")

log2Data = log2(sub.Data+1)

###heatmap plot

clust = pheno$Type3

names(clust) = pheno$SampleName

s.anno = data.frame(Dev.Time = factor(clust,levels=c("F45d","F100d","F157d","P5d","P5y"),

labels= c("F45d","F100d","F157d","Neonate","Adult") ) ,

Organ = as.factor( pheno$Type) )

cols = brewer.pal(n = 5, name = "Greens");

names(cols)= c("F45d","F100d","F157d","Neonate","Adult") ;

ano.col = list(Dev.Time = cols)

pheatmap(log2Data[,], cluster_cols = F, cluster_rows=F,border_color = NA,

gaps_row = c(8,24),gaps_col = c(14,26,40),

#color = colorRampPalette(brewer.pal(n = 9, name = "YlOrRd"))(20),

annotation= s.anno, annotation_colors = ano.col )

########################################################

#ggplots for lines

library(ggplot2)

library(ggpubr)

library(reshape2)

gg.dat = cbind(s.anno,Time = as.numeric(s.anno$Dev.Time), t(log2Data))

fil = gg.dat$Organ=="colon"| gg.dat$Organ =="lung"

#receptors

gg.gene = melt(gg.dat[fil,c(1:7) ], id = c("Dev.Time","Organ","Time"))

colnames(gg.gene)[4] = "Gene"

gg.gene$Organ = factor(gg.gene$Organ, levels = c("lung","colon"))

p1 = ggplot(data = gg.gene, aes(x = Time, y = value, color = Organ) ) +

geom_point() + stat_smooth(aes(fill = Organ),se = T, method="loess")+

scale_x_continuous(label = levels(gg.dat$Dev.Time)) +

theme_bw() + theme(axis.text.x = element_text(angle=45, hjust=1) )+

facet_grid(Organ ~Gene)+ ylab("log2(RPKM+1)")+ xlab("Development Time")

#ezmyes

gg.gene = melt(gg.dat[fil,c(1:3,13,19, 31,28) ], id = c("Dev.Time","Organ","Time"))

colnames(gg.gene)[4] = "Gene"

gg.gene$Organ = factor(gg.gene$Organ, levels = c("lung","colon"))

p2 = ggplot(data = gg.gene, aes(x = Time, y = value, color = Organ) ) +

geom_point() + stat_smooth(aes(fill = Organ),se = T, method="loess")+

scale_x_continuous(label = levels(gg.dat$Dev.Time)) +

theme_bw() + theme(axis.text.x = element_text(angle=45, hjust=1) )+

facet_grid(Organ ~Gene)+ ylab("log2(RPKM+1)")+ xlab("Development Time")

ggarrange(p1,p2, nrow = 2,labels = c("A","B"), common.legend = T)

######################correlation plot

library(corrplot)

par(mfrow = c(1,2))

par(mfrow = c(2,2))

organs = c("lung","colon","brain","liver")

for(organ in organs){

fil = gg.dat$Organ == organ

cor.dat = gg.dat[fil, c(4:7,13,19, 31,28)]

corr = cor(cor.dat)

corrplot(corr, addCoef.col = "grey",type = "lower")

rect(0.5,0.5,4.5,4.5, border = "red", lwd = 3)

}

#02######################for immune profiles MCPcounter

imm.mat = read.table("MCPcounter_immuneProfile.txt",

header=T, row.names=1, sep="\t", quote="")

imm.mat = data.matrix(imm.mat )

pheatmap(na.omit(imm.mat), show_rownames =T, show_colnames =T,

cluster_rows = F, cluster_cols = F,

gaps_col = c(14,26,40), border="grey90",

color = colorRampPalette(brewer.pal(n = 9, name = "YlOrRd"))(20),

annotation = s.anno, annotation_colors = ano.col )

#bar plot

gg.imm = cbind(s.anno,Time = as.numeric(s.anno$Dev.Time), t(imm.mat))

fil = gg.imm$Organ =="lung"

gg.bar = gg.imm[gg.imm$Time > 2 & fil, c(1:3, 4,5,7, 8,6)]

colnames(gg.bar)[4:ncol(gg.bar) ] = c("Tcells","CD8_Tcells","Bcells","NK_cells","Cytotoxic")

gg.bar = melt(gg.bar, id = c("Dev.Time","Organ","Time"))

gg.bar$Organ = factor(gg.bar$Organ, levels = c("lung","colon"))

colors = brewer.pal(n = 3, name = "Greens");

ggbarplot(data = gg.bar, x = "Dev.Time", y = "value",

xlab= "Development Stage", ylab="Expression Score",

ylim= c(0, 1.15* max(gg.bar$value)), facet.by = c("Organ","variable"),

fill = "Dev.Time", legend="right", palette = colors,

add = c("mean_se", "dotplot"),error.plot = "upper_errorbar")+

theme_bw()+ theme(axis.text.x=element_text(angle=45, hjust=1))+

stat_compare_means(aes(label = paste0("p = ", ..p.format..)),

comparisons = list(c("Adult","Neonate")), method="t.test")

##cytokines

cyto.mat = data.matrix(data[30:44,])

log2cyto =log2(cyto.mat+1)

pheatmap(log2cyto[,41:54],cluster_rows = F, cluster_cols = F,

annotation = s.anno[,], annotation_colors = ano.col )

#bar plot

gg.imm = cbind(s.anno,Time = as.numeric(s.anno$Dev.Time), t(cyto.mat ))

fil = gg.imm$Organ =="lung"

gg.bar = gg.imm[gg.imm$Time > 2 & fil, c(1:3,4,6,7)]

gg.bar = melt(gg.bar, id = c("Dev.Time","Organ","Time"))

gg.bar$Organ = factor(gg.bar$Organ, levels = c("lung","colon"))

colors = brewer.pal(n = 3, name = "Greens");

ggbarplot(data = gg.bar, x = "Dev.Time", y = "value",

xlab= "Development Stage", ylab="RPKM",

ylim= c(0, 1.15* max(gg.bar$value)), facet.by = c("Organ","variable"),

fill = "Dev.Time", legend="right", palette = colors,

add = c("mean_se", "dotplot"),error.plot = "upper_errorbar")+

theme_bw()+ theme(axis.text.x=element_text(angle=45, hjust=1))+

stat_compare_means(aes(label = paste0("p = ", ..p.format..)),

comparisons = list(c("Adult","Neonate")), method="t.test")

#end
